# Neuropsychological-EEG Activation in Genetic Generalized Epilepsy

**DOI:** 10.15844/pedneurbriefs-29-3-4

**Published:** 2015-03

**Authors:** J. Gordon Millichap, John J. Millichap

**Affiliations:** 1 Division of Neurology, Ann & Robert H. Lurie Children's Hospital of Chicago, Chicago, IL; 2 Departments of Pediatrics and Neurology, Northwestern University Feinberg School of Medicine, Chicago, IL

**Keywords:** Adolescence, Cognitive, Epileptiform discharges, Idiopathic generalized epilepsy, Neuropsychological activation

## Abstract

Investigators from Lithuanian University of Health Sciences, Kaunas, evaluated the effects of neuropsychological activation (NPA) tasks on epileptiform discharges (ED) in adolescents with idiopathic generalized epilepsy (IGE) and in comparison with hyperventilation and photic stimulation.

Investigators from Lithuanian University of Health Sciences, Kaunas, evaluated the effects of neuropsychological activation (NPA) tasks on epileptiform discharges (ED) in adolescents with idiopathic generalized epilepsy (IGE) and in comparison with hyperventilation and photic stimulation. Patients with IGE aged 14-17 years had a baseline video-EEG with habitual activation procedures followed by NPA tasks. The protocol of NPA included reading a Lithuanian fable, mentally and aloud, retelling the story, writing 4 sentences, mental and written calculation, and drawing a face. The tasks were classified as action-programming (written, calculation, drawing, reading, and writing), and thinking (mental calculation and mental reading). During at least one NPA task epileptiform EEG discharges were registered in 12 (20.3%) cases. None had a clinical seizure during tasks. Provocative effects of NPA tasks were more prevalent in photosensitive cases, especially action-programming NPA type (p = 0.04). The provocative effects of NPA were comparable to those of hyperventilation (23.7%) and photic stimulation (30.5%; p > 0.05). In the group of patients with ED on base-line EEG, no ED were registered during NPA in 14 (70%) out of 20 cases and the effect of NPA was regarded as inhibitory. NPA had no effect on 33 (84.6%) cases out of 39 without ED on base-line EEG. NPA is recommended in selected cases of IGE when routine EEG is not informative or when sleep EEG is not obtained. [1]

COMMENTARY. Matsuoka H of Tohoku University, Japan, has written extensively on neuropsychological EEG activation in patients with epilepsy [2]. In 480 patients with various epilepsies, NPA tasks provoked epileptic discharges in 38 (7.9%) patients, and were accompanied by myoclonic seizures in 15 patients, absence seizures in 8 and simple partial seizures in one. Mental activities associated with use of the hands i.e. writing (68.4%), written calculation (55.3%) and spatial construction (63.2%) provoked the most discharges followed by mental calculation (7.9%) and reading (5.3%). Action-programming type activities were most provocative in 32 of 38 (84.2%) patients, followed by thinking type activities in 4 (10.5%). Seizure-precipitating mental activities were almost always related to IGE and rarely (2 of 38 patients) related to temporal lobe epilepsy. In IGE patients, those with myoclonic seizures were more susceptible than absence or GTCS. Matsuoka considers the neuropsychological approach to epilepsy indispensable for assessment of cognitive function during pre- and postsurgical evaluation and for disclosing the semiology of nonconvulsive status epilepticus [3]. Whereas IGE patients with myoclonic seizures are vulnerable to higher mental activity, cognitive-motor function may have an inhibitory effect on EEG discharges in the majority of epilepsy patients [4].
